# Supplementary material for: Genome-Wide Urea Response in Rice Genotypes Contrasting for Nitrogen Use Efficiency
Source: Int J Mol Sci. 2023 Mar 23;24(7):6080. doi: 10.3390/ijms24076080 (PMC10093866; doi:10.3390/ijms24076080)
Supplement: Supplementary file 1 [file ijms-24-06080-s001.zip › ijms-1997633 supp 2.pdf]

Supporting information:

Supplementary Tables:

Supplementary Table S1: Nidhi and Panvel1 Urea DEGs

Supplementary Table S2: Gene Ontology analysis of Nidhi and Panvel1 DEGs

Supplementary Table S3: List of the primers used in this study.

Supplementary Table S4: Transporters of genotype Nidhi and Panvel1 encoded by DEGs, their genes, families, functions and references

Supplementary Table S5: Functional categorization of Transporters in Nidhi and Panvel1

Supplementary Table S6: Transcription Factors of genotype Nidhi and Panvel1 encoded by DEGs, their genes, families, functions and references

Supplementary Table S7: Functional categorization of Transcription factors in Nidhi and Panvel1

Supplementary Table S8: Conserved motifs in the promoter regions of Nidhi and Panvel1 urea DEGs, their TF family and gene ontology analysis

Supplementary Table S9: miRNAs of genotype Nidhi and Panvel1 encoded by DEGs, their target genes, functions and references

Supplementary Table S10: Details of protein-protein interaction (PPI) networks and their GO-based functional annotations

Supplementary Table S11: Details of urea-responsive PPI molecular complexes in Nidhi and Panvel1.

Supplementary Table S12: Details of the DEGs-associated post-translational modifications in Nidhi and Panvel1

Supplementary Table S13: DEGs encoding proteins for transporters and TFs

Supplementary TableS14: NUE-QTLs (from literature) and Nidhi urea NUE-genes colocalized onto NUE-QTLs

Supplementary Table S15: NUE-QTLs (from literature) and Panvel1 urea NUE-genes colocalized onto NUE-QTLs

Supplementary Table S16: Urea- and nitrate-responsive common and exclusive genes and associated pathways in Nidhi and Panvel1.

Supplementary Figures:

Supplementary Figure S1: Scatter plots show the correlation between the microarray data of Nidhi and Panvel1 grown under low (1.5 mM) and normal (15 mM) urea.

Supplementary Figure S2: Heatmap represents the expression pattern of urea regulated differentially expressed top two transporters and transcription factors' families for Nidhi and Panvel1 rice contrasting genotypes.

Supplementary Figure S3: DEGs associated protein-protein interaction (PPI) networks developed in Nidhi under low urea condition.

Supplementary Figure S4: DEGs associated protein-protein interaction (PPI) networks developed in Panvel1 under low urea condition.

Supplementary Figure S5: DEGs associated molecular complexes/subclusters detected in the protein-protein interaction networks developed in Nidhi.

Supplementary Figure S6: DEGs associated molecular complexes/subclusters detected in the protein-protein interaction networks developed in Panvel1.
