# Supplementary material for: Genome-Wide Urea Response in Rice Genotypes Contrasting for Nitrogen Use Efficiency
Source: Int J Mol Sci. 2023 Mar 23;24(7):6080. doi: 10.3390/ijms24076080 (PMC10093866; doi:10.3390/ijms24076080)
Supplement: Supplementary file 1 [file ijms-24-06080-s001.zip › Supplementary information/Supplementary Figure S1-S6.pdf]

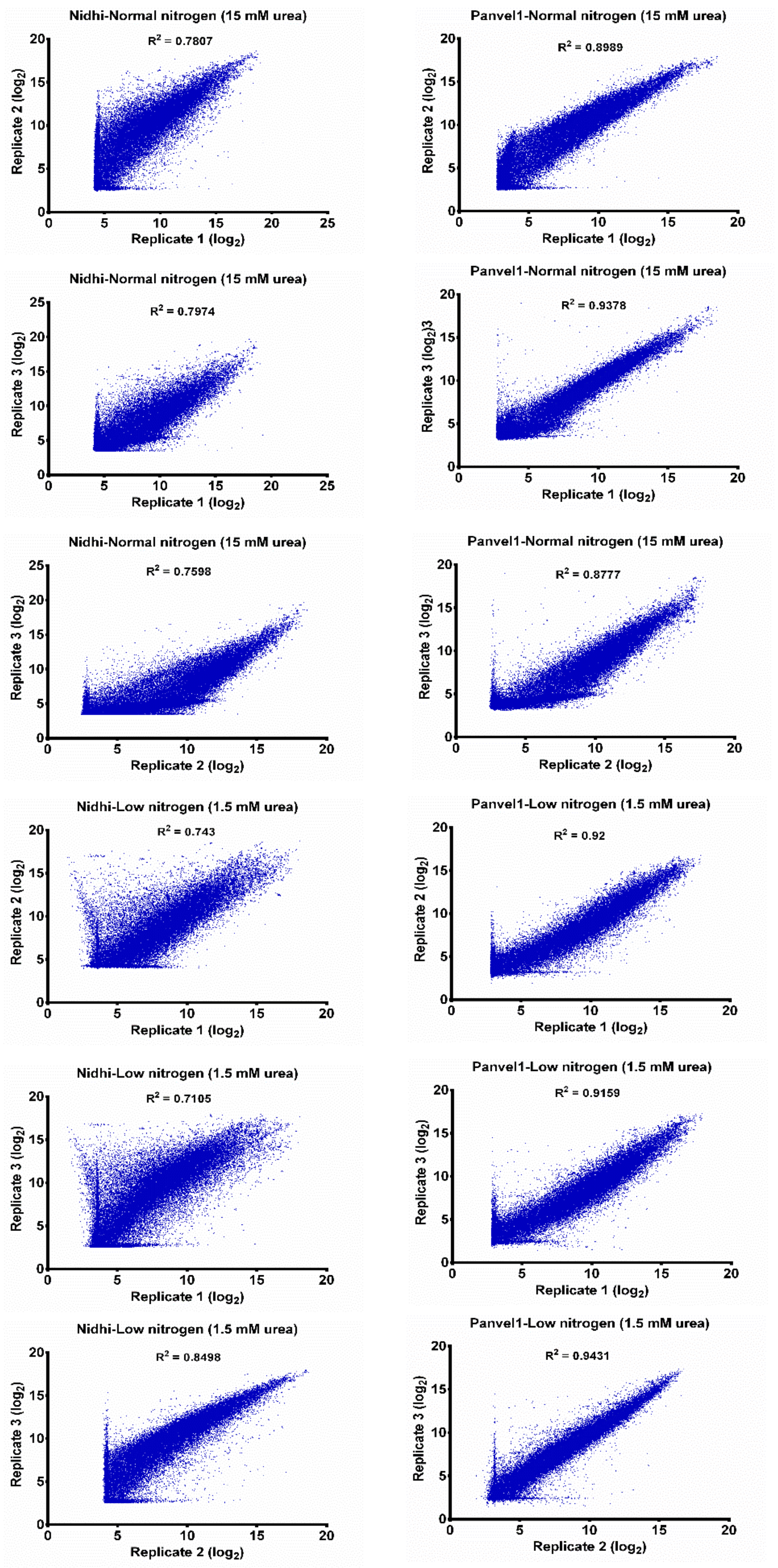

Figure S1: Scatter plots show the correlation between the microarray data of Nidhi and Panvel1 grown under low (1.5 mM urea) and normal (15 mM urea) nitrogen condition. Three independent biological replicates were used in this study.

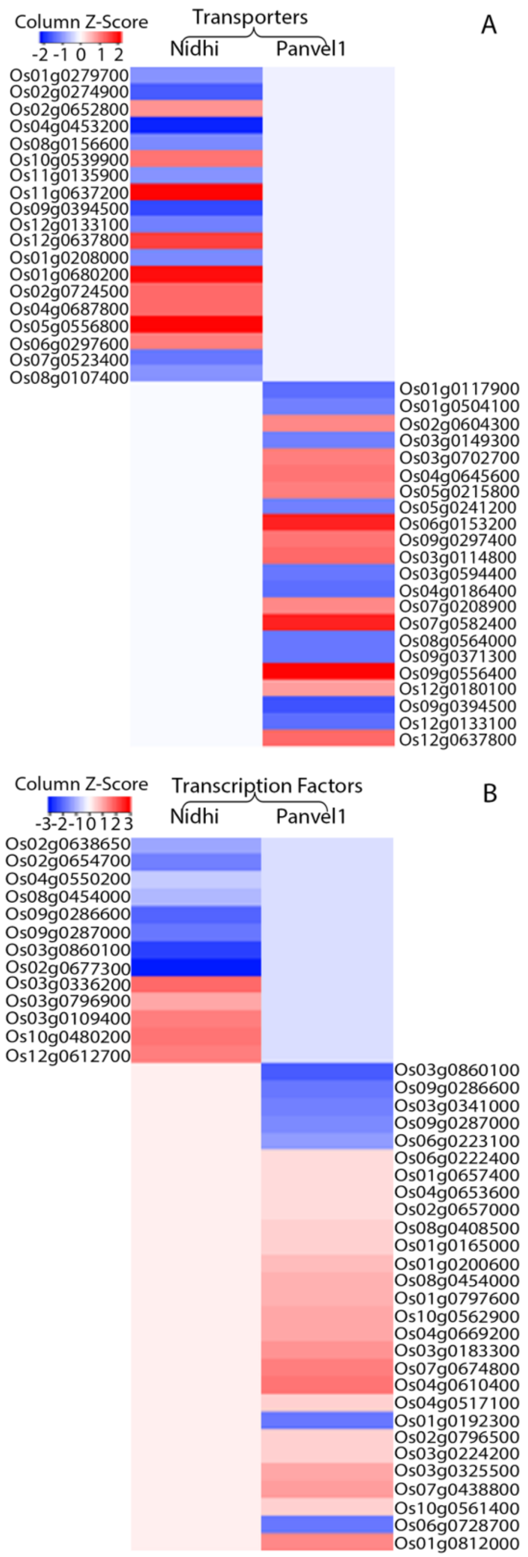

Figure S2: (A) Heatmap was constructed using Heatmapper, which represents the expression pattern of urea regulated differentially expressed genes (DEGs) of top two transporters' families for Nidhi and Panvel1 rice contrasting genotypes. The top two transporter families are Drug/Metabolite Transporter (DMT) and Major Facilitator Superfamily (MFS) for both the rice genotypes Nidhi and Panvel1. (B) Expression pattern of urea regulated DEGs of top two transcription factors' families for Nidhi and Panvel1 rice contrasting genotypes. For Nidhi the top two TFs families are AP2 and bZIP, while for Panvel1 they are AP2 and Myb/SANT.

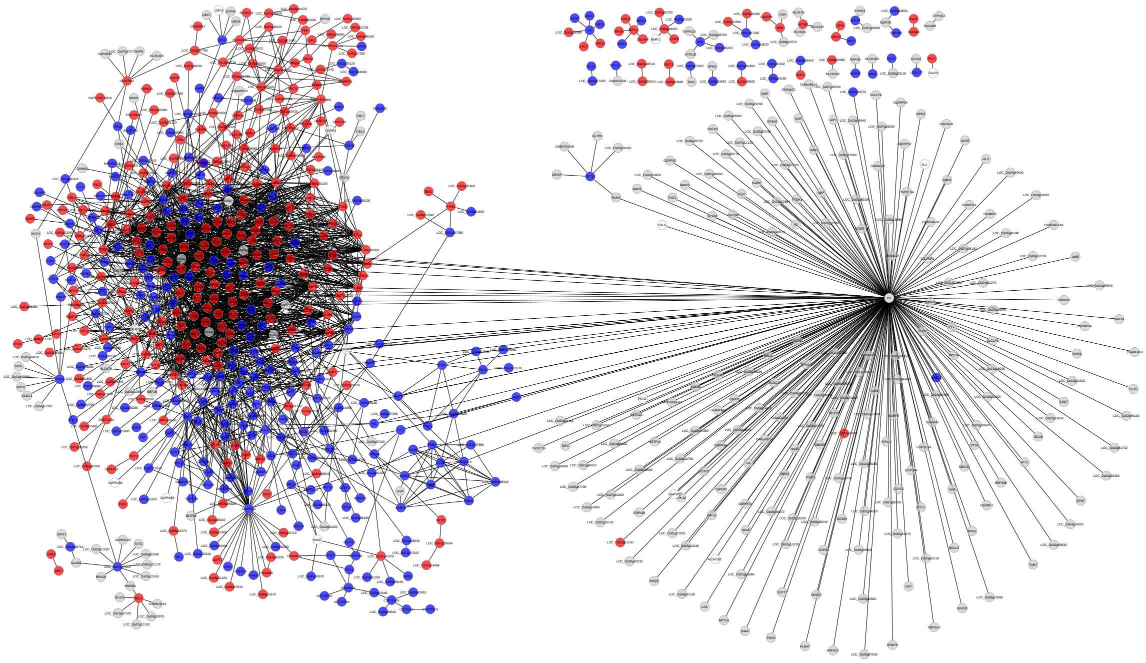

Figure S3: DEGs associated protein-protein interaction (PPI) networks developed in Nidhi under low urea condition. PPI networks were constructed in Cytoscape and expression value of DEGs were mapped onto the networks. Red and blue colour nodes represent the up- and down-regulated DEGs, respectively. Light colour nodes represent interactors, which are not DEGs in this study.

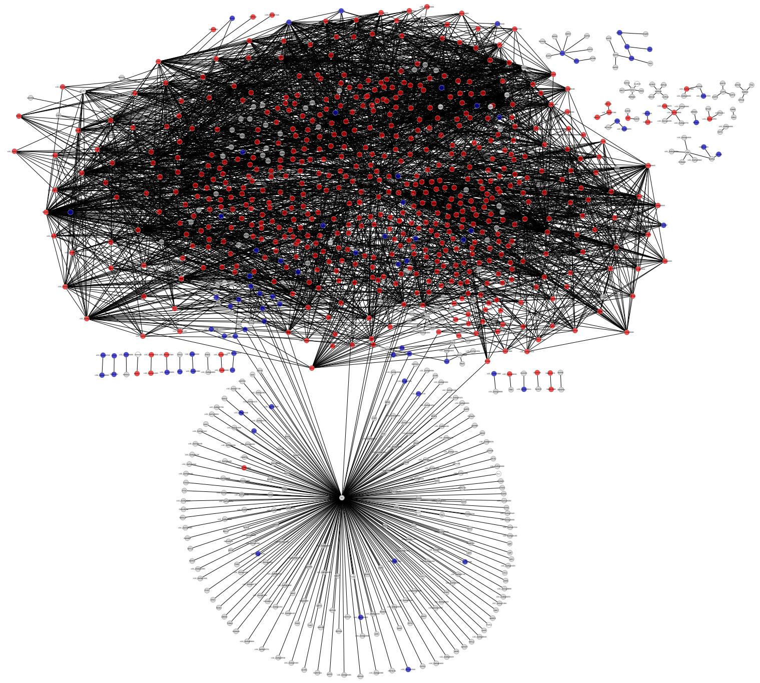

Figure S4: DEGs associated protein-protein interaction (PPI) networks developed in Panvel1 under low urea condition. PPI networks were constructed in Cytoscape and expression value of DEGs were mapped onto the networks. Red and blue colour nodes represent the up- and down-regulated DEGs, respectively. Light colour nodes represent interactors, which are not DEGs in this study.

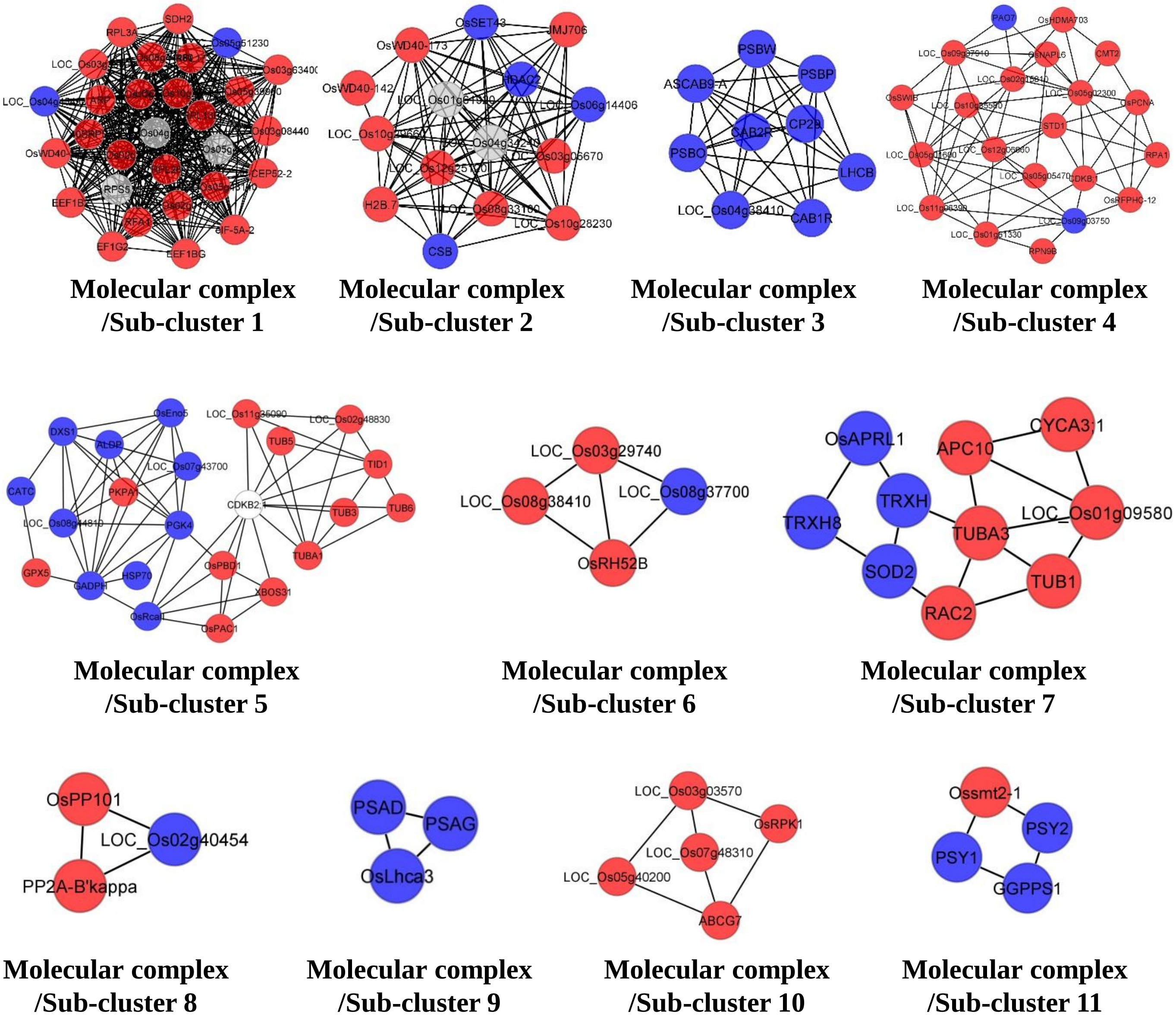

Figure S5: DEGs associated molecular complexes/subclusters detected in the protein-protein interaction networks developed in Nidhi. Red colour nodes represent the up-regulated DEGs, whereas blue colour nodes correspond to down-regulated DEGs. Non DEGs interactors are shown in light colour.

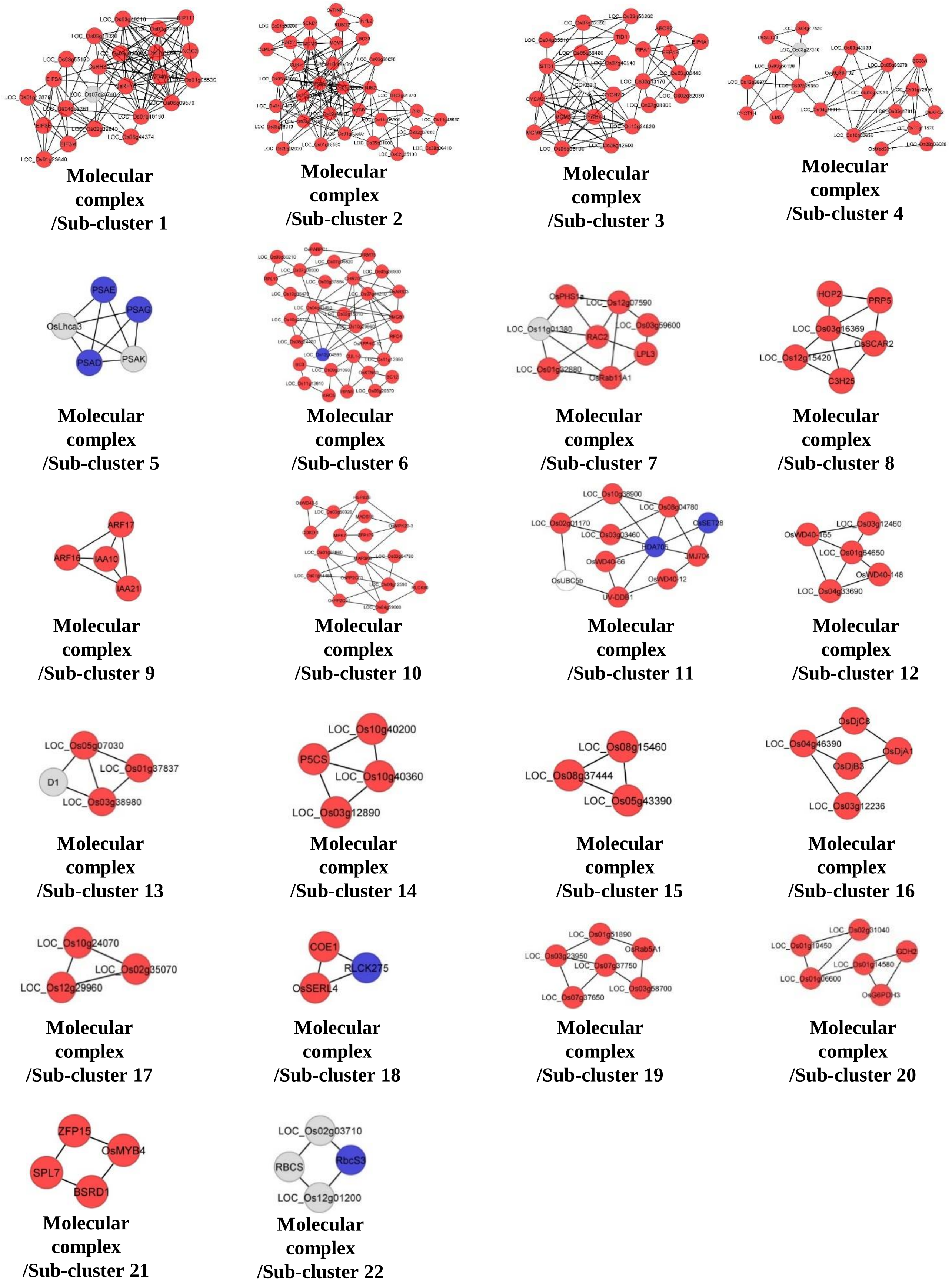

Figure S6: DEGs associated molecular complexes/subclusters detected in the protein-protein interaction networks developed in Panvel1. Red colour nodes represent the up-regulated DEGs, whereas blue colour nodes correspond to down-regulated DEGs. Non DEGs interactors are shown in light colour.
